# Biosafety Evaluation of a Chimeric Adenoviral Vector in Mini-Pigs: Insights into Immune Tolerance and Gene Therapy Potential

**DOI:** 10.3390/biomedicines12112568

**Published:** 2024-11-09

**Authors:** Andrei Izmailov, Irina Minyazeva, Vage Markosyan, Zufar Safiullov, Ilnaz Gazizov, Ilnur Salafutdinov, Maria Markelova, Ravil Garifulin, Maksim Shmarov, Denis Logunov, Rustem Islamov, Vadim Pospelov

**Affiliations:** 1 Department of Histology, Cytology and Embryology, Kazan State Medical University, 420012 Kazan, Russia; minyazevairina@yandex.ru (I.M.); ravil.l16rus@mail.ru (R.G.); 2 Department of Topographic Anatomy and Operative Surgery, Kazan State Medical University, 420012 Kazan, Russia; vage.markosyan@gmail.com; 3 Department of Anatomy, Kazan State Medical University, 420012 Kazan, Russia; redblackwhite@mail.ru (Z.S.); ilnazaziz@mail.ru (I.G.); 4 Institute of Fundamental Medicine and Biology, Kazan Federal University, 420008 Kazan, Russia; sal.ilnur@gmail.com (I.S.); mimarkelova@gmail.com (M.M.); 5 The National Research Center for Epidemiology and Microbiology Named after Honorary Academician N.F. Gamaleya of the Ministry of Health of the Russian Federation, 123098 Moscow, Russia; mmshmarov@gmail.com (M.S.); ldenisy@gmail.com (D.L.); 6 LLC “Impulse of Life”, Marshala Biryuzova Str., 32, 123060 Moscow, Russia; vadim_pospelov@mail.ru

**Keywords:** genetic vectors, adenoviruses, swine, immune tolerance, genetic therapy, biosafety, transcriptome, secretome, leucocytes, autologous genetically enriched leucoconcentrate

## Abstract

Background: The biosafety of gene therapy products remains a major challenge to their introduction into the clinic. In particular, the problem of immunogenicity of viral vectors is the focus of attention. Large animals such as pigs, whose anatomical and physiological characteristics are similar to those of humans, have an advantage in testing vector systems. Methods: We performed a comprehensive in vitro and in vivo study to evaluate the biosafety of a chimeric adenoviral vector carrying a green fluorescent protein gene (Ad5/35F-GFP) in a mini-pig model. Results: Transcriptome and secretome analyses of mini-pig leucocytes transduced with Ad5/35F-GFP revealed changes restraining pro-inflammatory processes and cytokine production. No adverse effects were revealed through the clinical, instrumental, laboratory, and histological examinations conducted within a week after the direct or autologous leucocyte-mediated administration of Ad5/35F-GFP to mini-pigs. The decrease in cytokine levels in the blood of experimental animals is also consistent with the in vitro data and confirms the immune tolerance of mini-pigs to Ad5/35F-GFP. Conclusions: Here, we show the safety of Ad5/35F in a mini-pig model and provide evidence that Ad5/35F is a promising vector for gene therapy. These results advance our understanding of vector–host interactions and offer a solid foundation for the clinical application of this vector.

## 1. Introduction

Gene therapy is a relatively novel and dynamically developing approach in the field of biomedicine. Currently, it remains a highly relevant area of study, particularly important for either the correction of mutated gene functions in inherited diseases [1,2] or the modulation of target cell functions through the expression of transgenes encoding biologically active molecules needed in specific therapeutic situations [3,4,5]. The delivery of transgenes to target cells can be achieved through both viral and non-viral (plasmid DNA, liposomes, exosomes, lipid nanoparticles, inorganic nanoparticles, cationic polymers, and polymer hydrogels) vector systems [6]. In clinical trials, viral delivery systems are utilized approximately four times more frequently than naked or plasmid DNA [7]. Recombinant viral replication-defective vectors derived from adenoviruses, adeno-associated viruses, herpes simplex viruses, retroviruses, and lentiviruses are widely employed for gene transfer in both in vivo and ex vivo gene therapies [8]. The nature of viral vector systems allows them to pass cell membranes via specific receptors, delivering genetic material to the nucleus, where it is transcribed into mRNA and subsequently resulting in ribosomal synthesis (transient or permanent) of recombinant proteins with therapeutic properties.

Direct (in vivo) gene therapy involves the systemic administration of a viral vector carrying a transgene, relying on the viral vector’s tropism and subsequent transduction of the target host cells [2]. In contrast, cell-mediated (ex vivo) gene therapy entails the delivery of transgenes using autologous or allogeneic cells as carriers, thereby avoiding direct interaction between viral antigens and host immunocompetent cells. This method ensures that transgenes are expressed solely by ex vivo transduced cells [5].

The choice between direct and cell-mediated gene therapies, as well as the appropriate vector system, is determined by the precise disease being treated [9]. Depending on whether the condition is inherited or acquired and whether temporary or lifelong production of the recombinant therapeutic molecules is required, treatment can target etiotropic, symptomatic, or pathogenetic aspects of the disease. However, the biosafety of both approaches remains a significant challenge in advancing gene therapy toward clinical application. Addressing the common side effects associated with viral vector systems is critical to improving the safety and efficacy of gene therapy [10,11].

In gene therapy, the selection of an appropriate delivery vector is crucial and must be considered alongside therapeutic genes, depending on the nature of the disease and the intended therapeutic outcomes, as was mentioned above. The choice of delivery vector is influenced by factors such as its packaging capacity (ability to encapsulate therapeutic cDNA), the duration of transgene expression (temporary or permanent), and biosafety concerns, including mutagenicity, immunogenicity, and toxicity [12]. Despite this, both viral and non-viral methods present specific advantages and disadvantages, making the selection of an optimal delivery vector an ongoing challenge [12,13,14]. Currently, there is limited understanding of the recipient organism’s response to vector systems (recombinant viruses) under in vivo and ex vivo gene therapy conditions [8].

Consequently, only a few gene therapy products have been approved for clinical use. For instance, in direct (in vivo) gene therapy, adenoviruses carrying the p53 gene are used for the treatment of head and neck squamous cell carcinoma, while adeno-associated viruses delivering the SMN1 gene are employed to treat spinal muscular atrophy (SMA) [15,16]. In cell-mediated (ex vivo) gene therapy, retroviruses expressing the ADA gene are used for treating severe combined immunodeficiency (SCID) [17], and lentiviruses are utilized to deliver the ABCD1 gene for the treatment of adrenoleukodystrophy (ALD) [18].

Currently, vectors based on human adenovirus serotype 5 (Ad5) are among the most frequently used vectors for transgene delivery into human cells [19] and are the most common in various clinical trials (∼26%) [20]. Adenoviruses contain double-stranded DNA and efficiently transduce a broad range of dividing and non-dividing cells through the coxsackie adenovirus receptors (CARs) present on the membranes of endothelial and epithelial cells, hepatocytes, and muscle cells [21]. Adenoviral vectors can accept transgenic inserts up to 7500 nucleotide pairs [22]. Because these vectors do not incorporate into the host cell genome, they remain in an extrachromosomal form, reducing the probability of changes in the expression of proto-oncogenes or anti-oncogenes, which lowers the risk of tumor development [23]. Furthermore, adenoviral vectors enable the rapid onset of transgene expression (within 1–2 days after delivery) but allow only transient expression, typically lasting for up to 3 weeks [24]. Adenoviruses can be produced at a titer exceeding 10^10^ PFU/mL, potentially enabling high levels of target gene expression in the target cells.

One disadvantage of Ad5-based vectors is their low transduction efficiency in CAR-deficient or CAR-negative cells, such as neuronal and hematopoietic cells. Thus, the absence or deficiency of CARs on these cell membranes limits the effectiveness of gene delivery using Ad5-based vectors. Tropism-modified Ad5 vectors were first developed in 1996 by Gall J. et al., who engineered an Ad5 vector expressing the Ad7 fiber, demonstrating a change in vector tropism [25]. A tropism-modified Ad5 vector with neuron-selective targeting properties was designed using the fiber knob domain from canine Ad serotype 2 (Ad5-CGW-CK2) [26]. Based on the fact that human Ad35 does not bind to CARs but recognizes CD46 (cluster of differentiation 46) [27], which is present on the surface of most nucleated human cells [28,29], the replacement of the Ad5 fiber with an Ad35 fiber was proposed for efficient transduction of hematopoietic cells. Such chimeric adenoviruses can efficiently transduce leucocytes [30] and human dendritic cells [31].

In our preclinical studies, we have developed a novel and potentially effective approach to personalized cell-mediated gene therapy designed for the temporary production of biologically active molecules aimed at correcting pathogenetic disease processes. We propose the use of autologous peripheral blood leucocytes (leucoconcentrate) as cellular carriers of recombinant cDNA encoding therapeutic molecules. To prepare autologous genetically enriched leucoconcentrate, we suggest employing a chimeric adenoviral vector (Ad5/35F) derived from human adenovirus serotype 5 with fibers from adenovirus serotype 35. These modified fibers exhibit a high affinity for CD46, which is expressed on all nucleated blood cells [32,33]. Additionally, we have established a transduction technology for leucoconcentrates within plastic blood containers, which eliminates the need for in vitro cultivation and avoids the use of animal-derived biological products, antibiotics, or other chemicals [34]. In recent studies using mini-pig models of ischemic stroke [35,36] and spinal cord injury [37], we demonstrated the beneficial effects of intravenously infused autologous leucoconcentrate simultaneously transduced with chimeric adenoviral vectors (Ad5/35F) carrying cDNA encoding human vascular endothelial growth factor 165 (VEGF165), human glial cell line-derived neurotrophic factor (GDNF), and human neural cell adhesion molecule 1 (NCAM1) on both the morphological and functional recovery of the brain and spinal cord.

The rapid advancements in translational research for neurological and psychiatric diseases [38] emphasize the importance of biosafety studies for novel, innovative therapies. We previously demonstrated that umbilical cord blood mononuclear cells transduced with Ad5 carrying cDNA encoding GFP or human VEGF165 efficiently express transgenes while maintaining transcriptome and secretome patterns. These findings support the biosafety of using newborn leucocytes and Ad5-based delivery vectors for cell-mediated gene therapy [39]. Translational research plays a critical role in bridging the gap between fundamental science and clinical practice [40,41]. Biosafety studies of novel gene therapy products in large animals with anatomical and physiological characteristics similar to humans further enhance the translational relevance of the findings, contributing significantly to gene therapy research. In this study, we conducted both in vitro and in vivo biosafety assessments of a chimeric adenoviral vector (Ad5/35F) carrying a reporter gene encoding a green fluorescent protein (GFP) in a mini-pig model.

## 2. Material and Methods

### 2.1. Study Design

The in vitro experiments involved transcriptome and secretome profiling of genetically modified leucocytes derived from mini-pig peripheral blood. A comparative biosafety evaluation of the chimeric adenoviral vector carrying the reporter transgene was performed in mini-pigs following either direct intravenous infusion or autologous leucocyte-mediated delivery. To evaluate the early response of the recipient organism, daily clinical examinations—including respiratory rate, oxygen saturation, blood pressure, pulse, and temperature—were conducted alongside behavioral assessments using the “open field” test and peripheral blood analysis (complete blood count and biochemical tests). These evaluations were performed daily for a week. On the 7th day of the experiment, histological examinations were conducted on organs from the immune, urinary, digestive, and cardiovascular systems.

### 2.2. Preparation of Chimeric Adenoviral Vector

In our previous studies, we employed a chimeric adenoviral vector based on human adenovirus serotype 5 with fibers derived from adenovirus serotype 35 (Ad5/35F) [30] to deliver therapeutic recombinant human genes into leucocytes. The incorporation of fibers from adenovirus serotype 35 enhances the transduction efficiency of leucocytes by targeting CD46 on the cell surface. This viral construct proved particularly effective for transducing leucoconcentrates prepared from peripheral blood directly within blood containers, eliminating the need for culturing equipment and additional reagents [42]. A chimeric recombinant, replication-defective adenoviral vector Ad5/35F was generated using Graham et al.’s method [43]. An Ad5/35F vector carrying the green fluorescent protein (GFP) gene was produced, as previously described [34,37]. HEK-293 cells were employed to propagate the recombinant Ad5/35F-GFP vector and to determine its titer using the plaque formation assay.

### 2.3. Preparation of the Autologous GFP-Enriched Leucoconcentrate

Genetically enriched leucoconcentrate (GEL) was prepared from 50 mL of mini-pig peripheral blood using Ad5/35F-GFP, following our original protocol [37,44]. Briefly, blood was collected from the v. subclavia into a plastic blood bag containing an anticoagulant–preservative solution (CPDA, Wego, Weihai, China). The leucoconcentrate was then prepared using the following steps: (1) the sedimentation of erythrocytes using 6% hydroxyethyl starch, followed by centrifugation and washing with saline to isolate the leucoconcentrate; (2) the transduction of leucocytes with Ad5/35F-GFP, conducted over 12 h by infusing Ad5/35F-GFP into the blood bag at a multiplicity of infection (MOI) of 10, calculated based on the leucocyte count in the leucoconcentrate and the Ad5/35F-GFP titer (3.0 × 10^10^ PFU/mL); and (3) washing with saline, centrifugation, and the subsequent removal of the supernatant. The remaining 30 mL of the solution in the bag constituted the genetically enriched leucoconcentrate carrying the GFP gene.

### 2.4. Cellular and Molecular Analysis of the GFP-Enriched Leucoconcentrate

Samples of native (non-transduced) leucoconcentrate and GFP-enriched leucoconcentrate were washed with DPBS buffer, and erythrocytes were lysed using a red blood cell (RBC) lysis solution (0.15 M ammonium chloride, 10 mM potassium bicarbonate, and 0.1 mM EDTA). Following rinsing with DPBS buffer, the processed leucocytes were seeded onto 10 cm untreated culture plates and incubated in an RPMI-1640 medium (PanEco, Moscow, Russia) supplemented with 10% fetal bovine serum (FBS; Biosera, Nuaille, France), 2 mM L-glutamine, penicillin (100 U/mL), and streptomycin (100 µg/mL) (PanEco, Moscow, Russia). The leucocytes were cultured in a humidified incubator at 37 °C with 5% CO_2_/95% air for 72 h. The cell growth and GFP synthesis in gene-modified leucocytes were monitored using an Axio Observer Z1 inverted fluorescence microscope (Carl Zeiss, Oberkochen, Germany) with phase contrast. For flow cell cytometry, RT-PCR, and RNA sequencing (RNAseq), the cells were harvested using 0.25% trypsin (Invitrogen, Waltham, MA, USA). The supernatants were collected for multiplex analysis of cytokines, chemokines, and growth factors.

#### 2.4.1. Flow Cytometry Analysis

The efficiency of leucoconcentrate transduction with Ad5/35F-GFP was analyzed using a FACS Aria III flow cytometer (BD Bioscience, New York, NY, USA) and BD FACS Diva7 software (BD Bioscience, New York, NY, USA) [45]. An argon laser was tuned to 488 nm, and fluorescent cells were detected using a 525 nm bandpass filter. Non-transfected cells served as a negative control to establish parameters for flow cytometric analysis. The data obtained from flow cytometry are expressed as the percentage of EGFP-positive cells.

#### 2.4.2. RT-PCR Assay

The total RNA was extracted from 1 × 10^6^ native and gene-modified leucocytes cultured for 72 h using the ExtractRNA (Evrogen, Moscow, Russia) standard TRIzol (Invitrogen, USA) RNA isolation method. RNA quantification was performed by measuring UV absorbance at 260 nm using an EzDrop 1000 spectrophotometer (Blue-Ray Biotech, New Taipei City, Taiwan). Reverse transcription of RNA was conducted according to the Mint cDNA Synthesis Mix kit instructions (Evrogen, Russia). cDNA was analyzed using the QuantGene 9600 Real-Time PCR System (Bioer, Hangzhou, China). Real-time PCR amplification conditions were as follows: 95 °C for 3 min, followed by 40 cycles of 95 °C for 10 s, 53 °C for 30 s, and 72 °C for 30 s, with a final extension at 72 °C for 2 min, including plate read. Each reaction was performed in triplicate in a total volume of 10 μL and contained 100 ng of diluted cDNA, 5X qPCRmix-HS SYBR (Evrogen, Russia), and 200 nM of each primer (see Table 1). mRNA expression levels were analyzed in triplicate and normalized to 18S ribosomal RNA. The results were expressed as relative gene expression using the 2^−ΔΔCt^ method [46,47].

#### 2.4.3. RNA Sequencing and Bioinformatics Analysis

Whole transcriptome profiling of the native leucocytes (6 individual samples) and gene-modified leucocytes transduced with Ad5/35F-GFP (6 individual samples) was carried out using the Illumina platform [48].

The total RNA was extracted from fresh frozen samples with TRIzol reagent (Thermo Fisher, Waltham, MA, USA), treated with DNase I (free of RNase) (NEB), and purified with the Quick-RNA isolation kit (Zymo Research, Irvine, CA, USA) according to the manufacturer’s instructions. Concentrations of the isolated RNA samples were assessed using a Qubit 2.0 fluorometer (Invitrogen). The RNA Integrity Number (RIN) of all RNA used was assessed on a 2100 Bioanalyzer (Agilent Technologies, Santa Clara, CA, USA) using the RNA 6000 Pico Kit (Agilent Technologies) and was greater than 8.

Samples with total RNA quantities of 800 ng were used for mRNA enrichment with the NEB Next Poly(A) mRNA Magnetic Isolation Module (NEB, #E7490S) kit (New England Biolabs, Ipswich, MA, USA). Libraries from the enriched mRNA were prepared using the NEBNext Ultra II Directional RNA Library Prep Kit and sample purification beads (NEB, #E7765S; New England Biolabs). Fragment library quality was assessed on the 2100 Bioanalyzer using a DNA High Sensitivity kit (Agilent, 5067-4626). The pooled DNA library was sequenced on the NextSeq 500 (Illumina, San Diego, CA, USA) using the 2 × 75 bp paired-end read mode. The base call files were converted to the FASTQ format using Bcl2Fastq (Illumina).

A total of 20,945,132 to 28,441,953 paired-end reads were generated per sample (average 25,132,744). The quality control of the reads was carried out using the FastQC software package (ver 0.12.1). The reads were pseudo-aligned to the porcine reference genome from the Ensembl database (Sscrofa11.1 [GCA_000003025.6]) using the Kallisto v.0.46 software [49]. Differentially expressed transcripts and genes were discovered using the R package “sleuth” (www.r-project.org, www.rdocumentation.org/packages/sleuth, accessed on 22 August 2024). The functional enrichment analysis of the differentially expressed genes from the Gene Ontology (GO) database was carried out using the WebGestalt (WEB-based Gene Set Analysis Toolkit https://www.webgestalt.org, accessed on 10 August 2024) [50] and g:Profiler (https://biit.cs.ut.ee/gprofiler/gost, accessed on 10 August 2024) [51]. The results of functional enrichment include significantly relevant GO terms (*p* < 0.05) representing biological processes, molecular functions, and cellular components.

#### 2.4.4. Multiplex Assay

The levels of cytokines, chemokines, and growth factors secreted by native and gene-modified leucocytes were measured using xMAP Luminex technology (Luminex, Austin, TX, USA) [52] with the MILLIPLEX Porcine Cytokine/Chemokine Magnetic Bead Panel, a 13-plex immunology assay (GM-CSF, IFNγ, IL-1α, IL-1β, IL-1ra, IL-2, IL-4, IL-6, IL-8, IL-10, IL-12, IL-18, and TNF-α) (Merck Millipore, Burlington, MA, USA), following the manufacturer’s instructions. Briefly, supernatants were collected 72 h after culturing native and gene-modified leucocytes and incubated with fluorescent beads for one hour. The beads were then washed and incubated with phycoerythrin–streptavidin for 10 min (Merck Millipore, Burlington, MA, USA). Analysis was performed using a Luminex 200 analyzer (Merck Millipore, Burlington, MA, USA) according to the manufacturer’s protocol. Analyte concentrations were determined using standard curves generated using Luminex IS 2.3 software (Millipore). Each sample was tested in triplicate.

### 2.5. Animals and Treatments

Mature Vietnamese pot-bellied mini-pigs (25–30 kg) of both sexes were housed individually in environments with controlled air conditioning, temperature, and light/dark cycles, with continuous access to food and water. All animal protocols were approved by the Kazan State Medical University Animal Care and Use Committee (approval No. 5 dated 26 May 2020) and were conducted in a humane manner to minimize animal suffering and the size of the experimental groups. The animals were used for in vivo studies of the early immune response (over a one-week period) to direct and cell-mediated intravenous infusions of Ad5/35F-GFP. This 7-day period was chosen to capture the development of the primary immune response.

#### 2.5.1. Experimental Groups

The animals were divided into two experimental groups based on the mode of delivery of the chimeric adenoviral vector carrying GFP (Ad5/35F-GFP). In the direct delivery group, 3 × 10^10^ PFU of Ad5/35F-GFP in 10 mL of 0.9% NaCl was infused via the auricular vein (in vivo group, *n* = 3). In the cell-mediated delivery group, 30 mL of autologous leucoconcentrate enriched with the GFP reporter gene was infused via the auricular vein (ex vivo group, *n* = 3). The number of viral particles used for the preparation of the genetically enriched leucoconcentrate (GEL) and for direct infusion was identical.

#### 2.5.2. Clinical and Laboratory Examination

General monitoring was performed before the administration of Ad5/35F-GFP and daily for a week every midday. This monitoring included the measurement of respiratory rate (RR), blood oxygen saturation (SpO_2_), blood pressure (BP), heart rate (HR), body temperature, and a behavioral “open field” test. The assessment of the animals’ general condition involved evaluating their reactions to auditory and tactile stimuli, body position within the enclosure, and appetite by measuring food consumption from the previous day. Body temperature was recorded using an electronic thermometer DT-501 (A&D, Beijing, China) via rectal measurement. The respiratory rate was assessed at the start of the clinical examination while the mini-pigs were calm. Oxygen saturation was measured using a veterinary pulse oximeter Zoomed UT100 (UTECH, Beijing, China) by placing the sensor on the tail tip. Heart rate and blood pressure were measured using a veterinary blood pressure monitor Zoomed OBM-ESM303-01 (OBM, Beijing, China) with a cuff placed on the upper forelimb.

In the open-field test, the mini-pigs were placed in a 3 m × 3 m arena subdivided into nine 1 m × 1 m squares by four red lines. The test, lasting 10 min, recorded the number of red lines crossed (indicative of horizontal locomotor activity), the duration of ball play in seconds (reflecting exploratory activity), and the number of defecation and urination events (indicating anxiety levels).

Blood samples were collected from the subclavian vein of each experimental animal before and one week after the intravenous infusion of Ad5/35F-GFP or autologous leucoconcentrate enriched with GFP. For complete blood count (CBC) analysis, blood was collected into seven vacuum tubes (K2 LIND-VAC 2 mL, 13 mm × 75 mm). For biochemical blood tests (BBTs), blood was collected into one vacuum tube (IMPROVACUTER 13 mm× 100 mm, 5 mL).

### 2.6. Histology and Immunofluorescence Analysis

#### 2.6.1. Samples Collection

Experimental animals were euthanized 7 days after the intravenous infusion of Ad5/35F-GFP or autologous leucoconcentrate enriched with GFP. Mini-pigs were anesthetized intramuscularly with Zoletil^®^ 100 (3 mg/kg; Virbac Sante Animale, Carros, France) and then connected to an inhalation anesthesia apparatus (Minor Vet Optima, Zoomed, Moscow, Russia). Anesthesia was maintained with a mixture of 2.0–2.5% isoflurane (Laboratorios Karizoo, S.A., Barcelona, Spain) in oxygen. Subsequently, potassium chloride (150 mg/kg) was administered via the auricular vein to ensure euthanasia. Following euthanasia, spleen, thymus, submandibular lymph nodes, liver, kidney, lung, heart, and bone marrow smears were harvested and processed for morphological examinations and fluorescence microscopy.

#### 2.6.2. Light Microscopy

For the morphological study, the collected organs were fixed in 4% paraformaldehyde (Sigma, St. Louis, MO, USA) in phosphate-buffered saline (pH 7.4) and then embedded in paraffin. Sections of 5 µm thickness were prepared using a manual rotary microtome (HM 325; Thermo Scientific, USA), mounted on slides, and stained with hematoxylin and eosin for light microscopy.

#### 2.6.3. Fluorescent Microscopy

To assess GFP production in the organs, the samples were embedded in a tissue-freezing medium (Electron Microscopy Sciences, Hatfield, PA, USA), and 10 µm thick frozen sections were prepared using a cryostat (Microm HM 560; Thermo Scientific, Waltham, MA, USA). Sections for visualization of cell nuclei were counterstained with DAPI (10 µg/mL in PBS; Sigma), embedded in glycerol (GalenoPharm, Saint Petersburg, Russia), and examined using a fluorescence microscope (Axioscope A1; Carl Zeiss, Oberkochen, Germany).

### 2.7. Statistics

Processing, analysis, and visualization of the data were conducted using R 4.4.1 (R Foundation for Statistical Computing, Vienna, Austria). Descriptive statistics are reported as means ± standard deviations for multiplex analysis, behavioral assessments, instrumental evaluations, and blood tests. Statistical significance was assessed using the Kruskal–Wallis test with a threshold of *p* < 0.05. The results were visualized using tables, histograms, and heatmaps. Details of the RT-PCR and bioinformatics analysis are provided in the corresponding sections.

## 3. Results

### 3.1. In Vitro Study

#### 3.1.1. Expression of GFP in Gene-Modified Leucocytes

**Figure 1:**
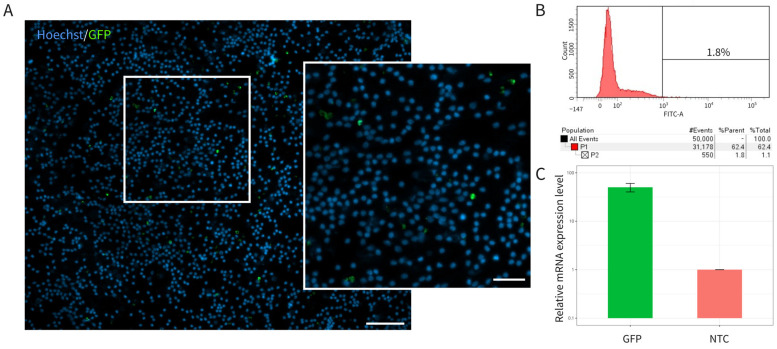
Expression of the reporter green fluorescent protein (GFP) gene by mini-pig leucocytes 72 h after transduction with Ad5/35F-GFP (MOI = 10). (**A**) Fluorescent microscopy showing GFP-positive leucocytes (green fluorescence) with cell nuclei stained with Hoechst 33342 (blue fluorescence). Scale bar = 100 µm; scale bar in the insert = 50 µm. (**B**) Flow cytometry analysis indicating the proportion of GFP-positive leucocytes. (**C**) qRT-PCR analysis of *GFP* mRNA levels in native (non-transduced, NTC) and gene-modified (GFP) leucocytes.

The efficiency of leucocyte transduction by Ad5/35F-GFP was confirmed 72 h after culturing the gene-modified cells. Fluorescence microscopy revealed a distinct green fluorescence in the cytoplasm of the gene-modified leucocytes (Figure 1A). Flow cytometry analysis showed that 1.8% of leucocytes were GFP-positive at a multiplicity of infection (MOI) of 10 (Figure 1B). Quantitative RT-PCR further confirmed *GFP* expression, with the mRNA level in gene-modified leucocytes being increased 50-fold compared to non-transduced cells (Figure 1C).

Thus, the transduction of mini-pig leucocytes with Ad5/35F-GFP at an MOI of 10 resulted in the effective expression of *GFP* at both mRNA and protein levels, with 1.8% of GFP-positive leucocytes detected.

#### 3.1.2. Multiplex Analysis of the Gene-Modified Leucocytes Secretome

**Figure 2:**
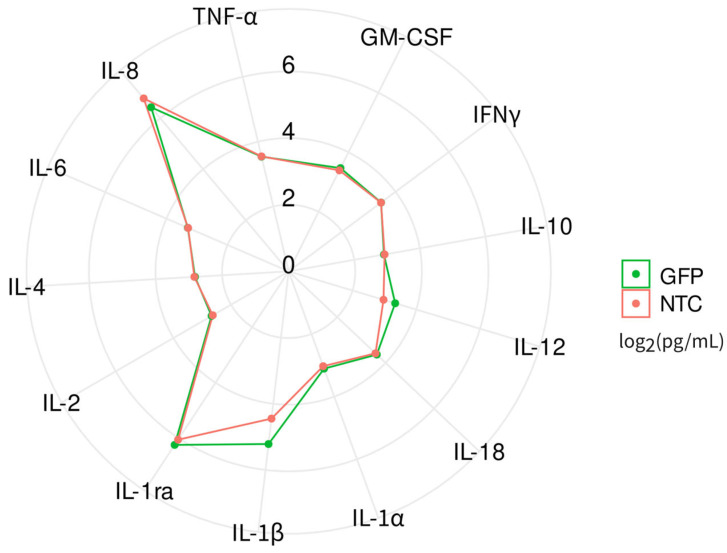
Radial comparative diagram of cytokine, chemokines, and growth factors in supernatants collected 72 h after the incubation of native (non-transduced, NTC) and gene-modified (GFP) leucocytes. Each sample was carried out in three technical repeats.

Simultaneously, the levels of 13 analytes were assessed in supernatants collected 72 h after culturing gene-modified leucocytes (Figure 2. Evaluation of experimental samples (*n* = 5) and control samples (from non-transduced cells, *n* = 5) revealed no significant differences in the levels of the studied analytes between the groups.

In summary, secretome analysis demonstrated that the chimeric adenoviral vector does not alter the production of the studied cytokines, chemokines, or growth factors in genetically modified mini-pig leucocytes.

#### 3.1.3. Analysis of the Gene-Modified Leucocytes Transcriptome

**Figure 3:**
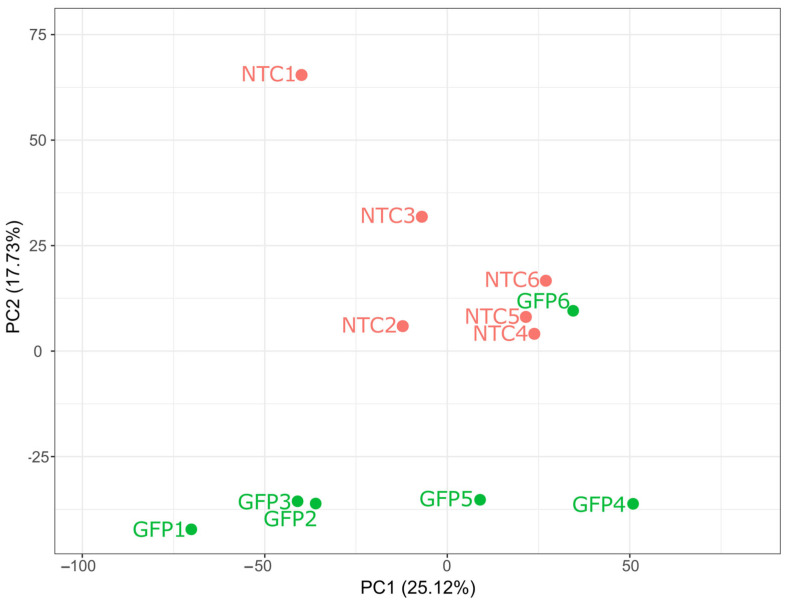
Principal component analysis and hierarchical clustering. The observations belonging to the two experimental groups are distributed in the space of the first two principal components, explaining 42.85% of the variance. PC1—principal component 1, PC2—principal component 2. The percentage values in the axis labels represent the percentage of variation described by this component. NTC1–6—native leucocytes (6 individual samples), GFP1–6—gene-modified leucocytes transduced with Ad5/35F-GFP (6 individual samples).

Transcriptome profiles of native, non-transduced cells (NTC, *n* = 6) and Ad5/35F-GFP-modified leucocytes (GFP group, *n* = 6) from individual animal blood samples were analyzed using 12 cDNA libraries. RNA-seq data identified transcripts for 19,210 genes. Quality control metrics confirmed high sequencing fidelity, with an average read alignment rate exceeding 88.8% (93% on average). Principal component analysis (PCA) demonstrated distinct differences in the overall gene expression profiles between native and gene-modified leucocytes. Variability among individual samples within the comparison groups was observed (Figure 3).

**Figure 4:**
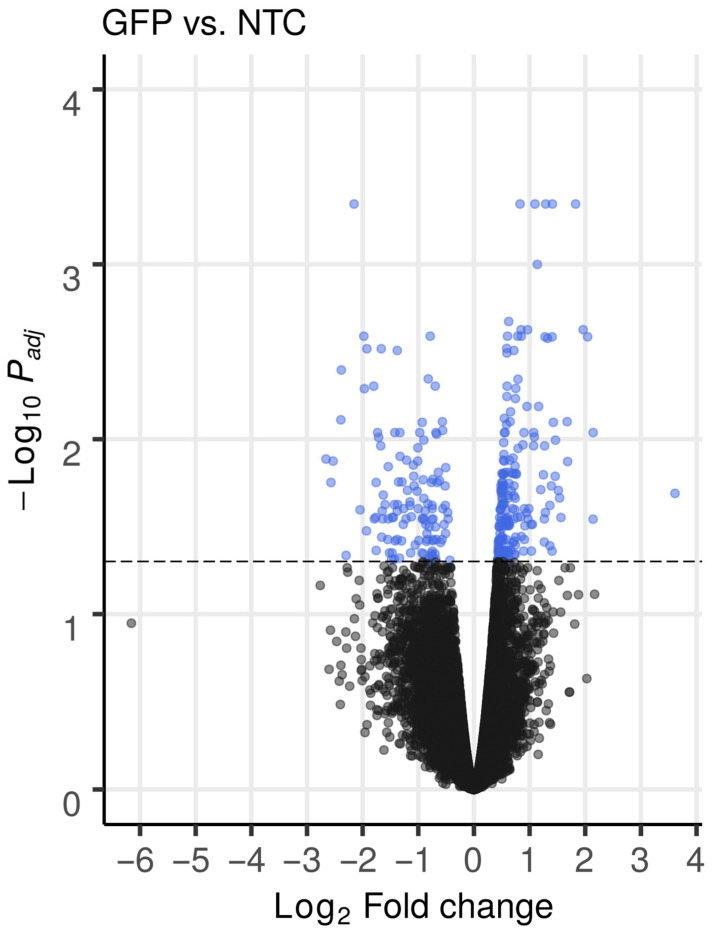
Volcano plot presents the differential performance of gene expression in non-transduced and gene-modified leucocytes. Sets of genes considered as differentially expressed using declared cutoff rules (pFDR-value (q-value) < 0.05) are marked as blue dots and dotted horizontal line.

**Figure 5:**
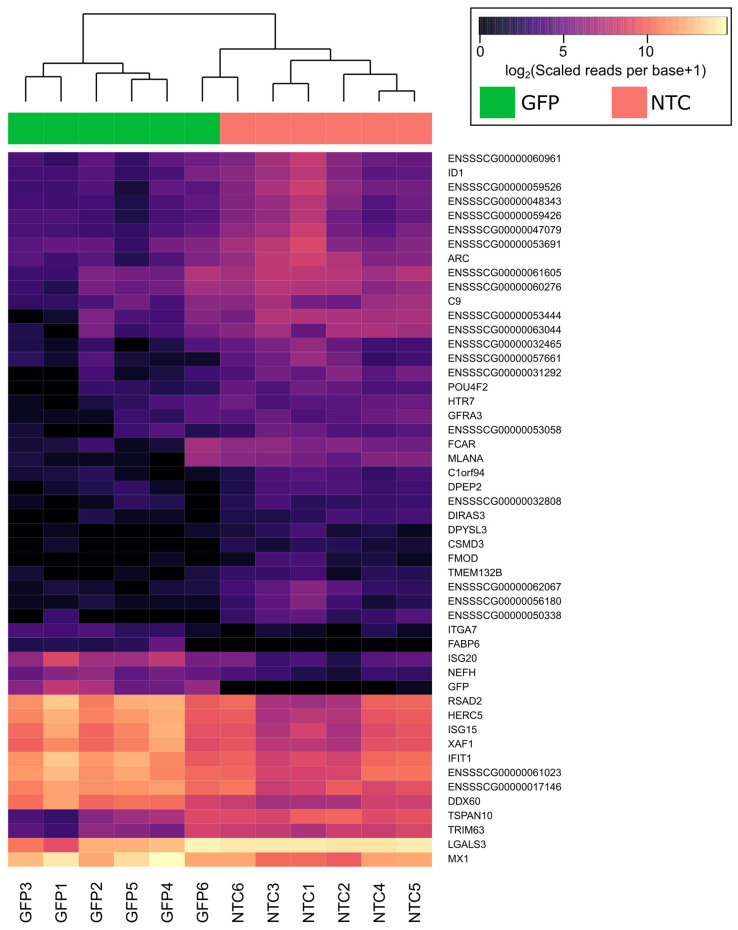
Heatmap of top 50 differentially expressed genes after hierarchical clustering. The reporter green fluorescent protein (GFP) gene is also on the list. NTC1–6—native leucocytes (6 individual samples), GFP1–6—gene-modified leucocytes transduced with Ad5/35F-GFP (6 individual samples).

Analysis of differential expression between NTC and GFP groups revealed 122 downregulated and 195 upregulated genes (Figure 4). In all samples from the GFP group, the expression of the *GFP* gene increased more than 12-fold compared to the NTC group. The list of genes considered as differentially expressed is presented in Supplementary Material (Table S1). The top 50 differentially expressed genes after hierarchical biclustering are shown in Figure 5.

**Figure 6:**
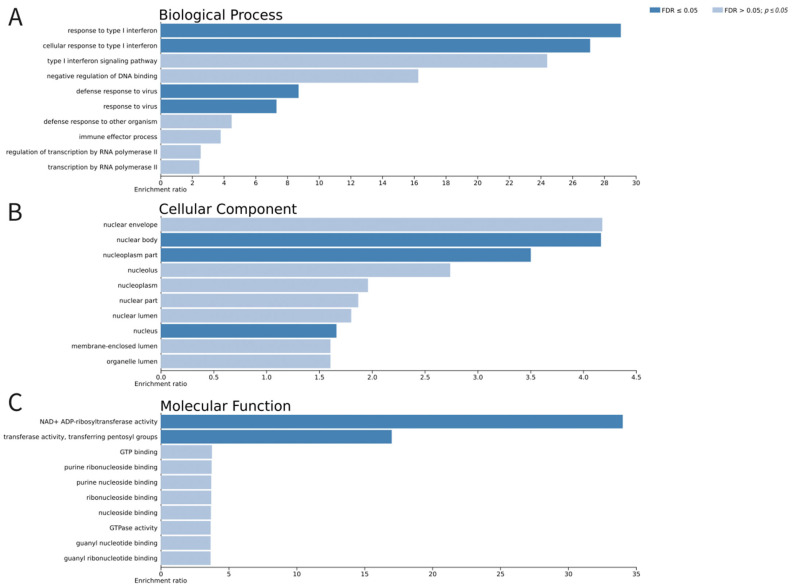
Gene Ontology-based (GO) enrichment analysis of differentially expressed genes in the non-transduced (NTC) and gene-modified (GFP) leucocytes. Enriched GO terms include biological processes (**A**), cellular components (**B**) and molecular functions (**C**).

Functional evaluation of the differentially expressed genes was performed based on Gene Ontology-based (GO) enrichment analysis, which allows for the discrimination of biological processes, molecular functions, and cellular components that are possibly affected after transduction of the leucocytes with Ad5/35F-GFP. A GO-based enrichment analysis of differentially expressed genes in the category of biological processes highlighted pathways connected with response to the virus. The majority of the genes associated with the category of cellular components were related to a nucleus. In the category of molecular functions, differentially expressed genes were involved in ribonucleoside binding. The results of the functional evaluation of the differentially expressed genes are demonstrated in Figure 6 based on WebGestalt analysis and in Supplementary Material based on g:Profiler (Table S2). Discovered transcriptomic changes suggest that the gene modification not only enhanced specific target gene expression but also triggered broader changes in cellular signaling.

Comprehensive bioinformatics analysis of the transcriptome in genetically modified mini-pig leucocytes, with 19,210 differentially expressed genes identified, revealed GO-based terms related to the immune response to Ad5/35F-GFP, such as GO:0051607 (defense response to virus) and GO:0071357 (cellular response to type I interferon), which may be responsible for inhibiting pro-inflammatory pathways and cytokine production, as shown above in the multiplex analysis of the gene-modified leucocytes secretome.

### 3.2. In Vivo Study

#### 3.2.1. Behavioral and Instrumental Tests

**Figure 7:**
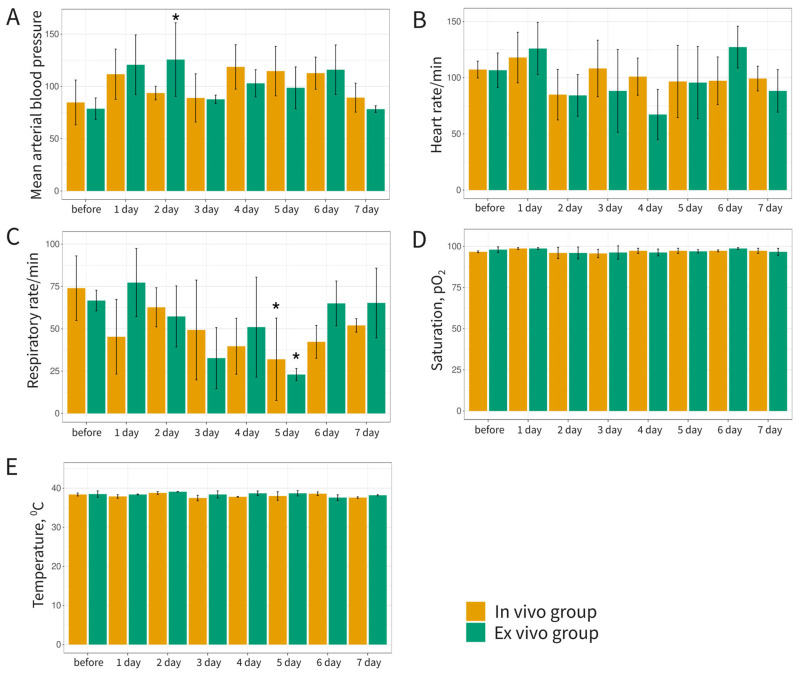
Results of everyday instrumental examination of blood pressure (**A**), heart rate (**B**), respiratory rate (**C**), blood oxygen saturation (**D**), and body temperature (**E**) in mini-pigs before and after direct (in vivo group) and cell-mediated (ex vivo group) delivery of Ad5/35F-GFP. Each measurement was performed in duplicate, * *p* < 0.05.

In the open-field test, mini-pigs from both experimental groups showed no significant differences in horizontal locomotor activity and exploratory behavior (Table 2). The absence or infrequent instances of defecation and urination across all animals indicated minimal anxiety during the test. Instrumental measurements of blood pressure (Figure 7A), heart rate (Figure 7B), respiratory rate (number of breaths per minute) (Figure 7C), blood oxygen saturation (Figure 7D), and body temperature (Figure 7E) revealed no notable changes following the administration of Ad5/35F-GFP, whether in vivo or ex vivo.

Comparative analysis of behavioral and instrumental data collected from both groups of animals within a week of Ad5/35F-GFP administration indicated no adverse effects.

#### 3.2.2. Blood Tests

Complete blood count analysis revealed a tendency toward decreased total leukocyte levels in both experimental groups 7 days after Ad5/35F-GFP delivery, relative to baseline measurements. However, these values remained within normal limits [53]. This decrease was consistent with a reduction in neutrophil counts, while lymphocyte levels remained stable and within the normal range (Table 3).

Biochemical assays assessing liver and kidney function—including alanine aminotransferase (ALT), total bilirubin, total protein, albumin, globulin, urea, creatinine, calcium, sodium, potassium, and chloride—as well as glucose levels, showed no significant changes and remained within normal ranges in both groups at the end of the experiment (Table 3).

**Figure 8:**
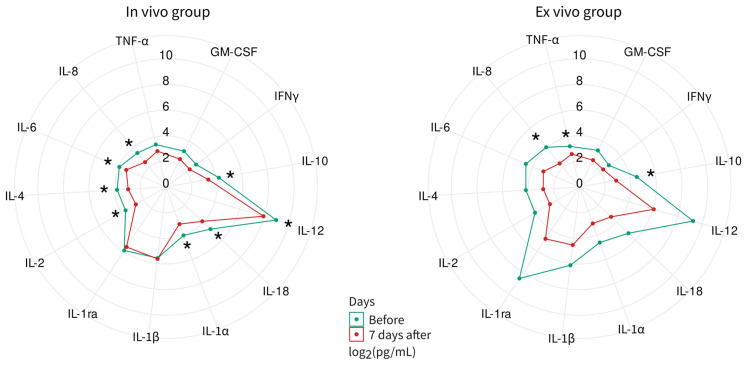
Radial comparative diagram of cytokine, chemokines, and growth factors in blood collected from mini-pigs before and 7 days after direct (in vivo group) and cell-mediated (ex vivo group) delivery of Ad5/35F-GFP. Each sample was carried out in three technical repeats. * *p* < 0.05.

Multiplex analysis of 13 analytes in the blood serum of animals from the direct delivery (in vivo group) and cell-mediated delivery (ex vivo group) of Ad5/35F-GFP revealed variations in analyte levels measured before and 7 days after Ad5/35F-GFP administration (Figure 8). In the in vivo group, levels of IL-1α, IL-2, IL-4, IL-6, IL-8, IL-10, IL-12, and IL-18 decreased. In the ex vivo group, decreases were observed in IL-10, IL-18, and TNF-α.

Hematological parameters showed a moderate response to Ad5/35F-GFP administration, particularly leukopenia resulting from a reduction in neutrophil count. However, biochemical parameters did not indicate any cytotoxicity. The observed decrease in certain blood cytokine levels in both groups of animals aligns with the transcriptomic and secretomic data obtained from the in vitro study.

#### 3.2.3. Histology Study

**Figure 9:**
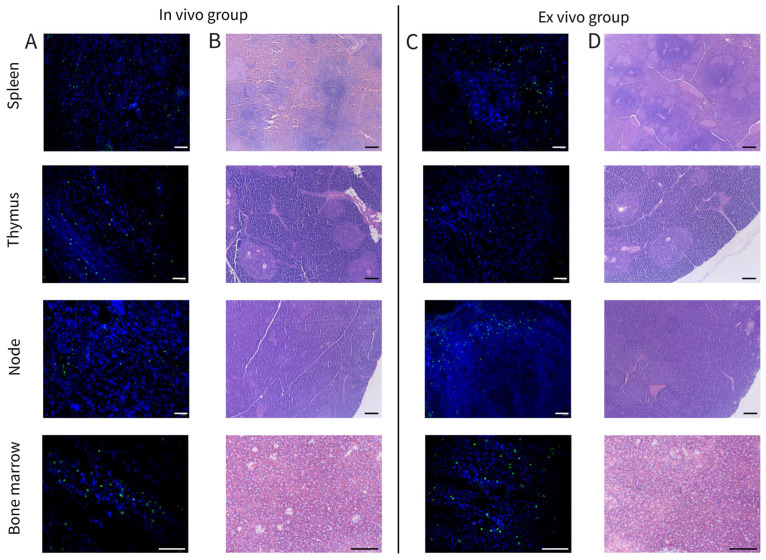
Fluorescent (**A**,**C**) and morphological (**B**,**D**) examination of the spleen, thymus, submandibular lymph nodes, and bone marrow in the mini-pigs after 7 days of direct (in vivo group) and cell-mediated (ex vivo group) delivery of Ad5/35F-GFP. (**A**) In situ transduced cells and (**C**) transplanted gene-modified leucocytes demonstrate the green glow. Nuclei counterstained with DAPI show the blue glow. Scale bar in (**A**,**C**) = 50 µm; scale bar in (**B**,**D**) = 200 µm.

Fluorescent microscopy revealed GFP-positive cells in the spleen, thymus, submandibular lymph nodes, and bone marrow following both in vivo and ex vivo delivery of Ad5/35F-GFP (Figure 9A,C). No GFP-specific green fluorescence was observed in the heart, lung, liver, or kidney. Morphological examination of the immunocompetent organs showed only moderate changes in the lymph nodes in both the in vivo and ex vivo groups (Figure 9B,D). These changes included signs of paracortical hyperplasia with a disrupted pattern of lymphoid follicles and sinus histiocytosis. No pathological changes were detected in the heart, lung, liver, or kidney in either group.

Histological examination revealed no pathological changes in parenchymal organs, except for a mild reaction to Ad5/35F-GFP in the lymph nodes of mini-pigs from both groups, which is consistent with the results of blood biochemical analysis. The absence of GFP-positive cells in parenchymal organs suggests low sensitivity of these cells to Ad5/35F following in vivo administration of Ad5/35F-GFP, as well as limited homing of gene-modified autologous leucocytes into intact organs after ex vivo administration.

Thus, we performed a comprehensive in vitro and in vivo study to evaluate the biosafety of the chimeric adenoviral vector with modified fibers carrying a reporter transgene encoding GFP. In the vitro studies, the efficacy of the Vietnamese pot-bellied mini-pigs leucocytes transduction with Ad5/35F-GFP was confirmed by transgene expression at mRNA and protein levels. Transcriptome bioinformatics analysis of the gene-modified cells discovered GO-based biological processes associated with the changes restraining pro-inflammatory pathways and cytokine production. The data on secretome profiling (production of cytokines, chemokines, and growth factors) did not reveal the difference between gene-modified and non-transduced leucocytes. In the in vivo study, it was shown that no side effects (clinical observation, instrumental analysis, and blood tests) due to activation of the immune system were observed 7 days after the direct intravenous injection of Ad5/F35-GFP or intravenous infusion of autologous leucoconcentrate transduced with Ad5/F35-GFP. No pathomorphological changes were found in the heart, lung, liver, and kidney of both groups. Demonstration of the GFP-positive cells in the spleen, thymus, submandibular lymph nodes, and bone marrow was accompanied by the decreased level of the studied cytokines in the blood of the animals after in vivo or ex vivo delivery of Ad5/35-GFP.

## 4. Discussion

In the short time since its first report in 1989, gene therapy has made significant strides in biomedicine worldwide. Thousands of clinical trials have been conducted, and a wide range of gene products have been developed; however, only a few gene therapy products have been approved for clinical use [54]. Despite improvements in therapeutic gene transfer using viral and non-viral technologies, the translation of biomedical research into clinical medicine remains limited. Non-viral vector-based transgene delivery is relatively inefficient compared to viral vectors, which, despite safety concerns, remains the most commonly used approach. The safety concerns surrounding viral systems arise from the natural potential of viruses for cytotoxicity, mutagenicity, and immunogenicity in host organisms. While these factors vary depending on the different virus types and their genetic modification into viral vectors can reduce some of their negative impacts, the biosafety of viral vectors remains a critical issue, slowing the approval of new gene therapy products.

Adenoviral systems are widely applied in gene therapy research, particularly for achieving transient gene expression, which provides a cost-effective means of delivering recombinant therapeutic proteins [55]. Adenoviral vectors can be administered via two primary routes: direct injection and cell-mediated delivery. Direct gene therapy, while straightforward, has notable drawbacks, including significant immunogenicity and uncontrolled transduction of various cell types throughout the body [19]. Additionally, systemic administration of adenoviral vectors can lead to cytotoxic effects on infected cells. Cell-mediated delivery addresses some of these issues but introduces challenges in selecting an appropriate cellular vehicle [14]. We have recently developed an innovative approach to transient gene therapy using a patient’s leucocytes derived from peripheral blood and transducing them with a chimeric adenoviral vector (Ad5/35F). This vector effectively targets leucocytes via the CD46 receptor [27,30]. Given the ability of leucocytes to circulate in vessels throughout the body, exit the bloodstream into nearly all tissues of the organism (especially in pathological conditions), and exhibit high secretory activity, it indicates the reasonability of using autologous leucocytes as cell carriers for therapeutic genes and effective producers of recombinant molecules that exert their effects on target cells through endocrine or paracrine mechanisms. Building on our previous studies demonstrating the therapeutic efficacy of an autologous leucoconcentrate enriched with three recombinant genes encoding VEGF165, GDNF, and NCAM1, using Ad5/35F, in treating mini-pigs with ischemic stroke [35,36] and spinal cord injury [37] we now report the biosafety evaluation of this chimeric adenoviral vector in vitro and in vivo studies in mini-pigs.

Swine are the exclusive natural hosts for porcine adenoviruses (PAdVs), which are classified into three groups: PAdV-A (serotypes 1–3), PAdV-B (serotype 4), and PAdV-C (serotype 5). In domesticated pigs, PAdV typically acts as a low-grade pathogen, often causing subclinical infections, such as mild, short-term diarrhea. It is commonly isolated from the gastrointestinal tracts of healthy pigs without associated clinical symptoms [56]. Due to the low immunogenicity of wild-type PAdVs, recombinant versions are frequently employed in vaccine development for swine infections [57]. Thus, the rationality of translational study on domestic swine of the adenoviral vectors used for vaccination and gene therapy in humans may be useful in terms of pig’s tolerance to adenoviral antigens and investigation of the direct therapeutic effects of transgenes.

In our in vitro study, we confirmed the transduction of mini-pig leucocytes with Ad5/35F-GFP at a multiplicity of infection (MOI) of 10 at both the transcriptional and protein synthesis levels. However, flow cytometry revealed that only 1.8% of the leucocytes were GFP-positive. A multiplex analysis of the secretome of leucocytes transduced with Ad5/35F-GFP demonstrated that the chimeric adenoviral vector did not affect the secretion of the cytokines, chemokines, and growth factors studied. These findings are consistent with previous research on human umbilical cord blood mononuclear cells transduced with Ad5-GFP [39]. Comparative transcriptome analysis of native and gene-modified leucocytes identified 122 downregulated and 195 upregulated genes out of 19,210 differentially expressed genes. Bioinformatics analysis of the transcriptomes discovered GO-based biological processes, which are semantically associated with terms related to the immune response to viral infection, such as GO:0051607 (defense response to virus) and GO:0071357 (cellular response to type I interferon), which may play a role in modulating pro-inflammatory pathways and cytokine production [58]. These results suggest that leucocytes from miniature Vietnamese pot-bellied pigs exhibit low sensitivity to the Ad5/35F chimeric adenoviral vector, as evidenced by the low transduction rate and the observed transcriptome and secretome profiles.

The early response to the intravenous administration of Ad5/35F-GFP, either in vivo or ex vivo, was evaluated in mini-pigs over the first seven days of the experiment. Clinical observations and instrumental measurements remained stable throughout the week after Ad5/35F-GFP administration in both experimental groups. On day 7, biochemical liver and kidney function tests were within normal limits for both the in vivo- and ex vivo-treated mini-pigs. However, evidence of an immune response to the chimeric adenoviral vector was noted in the complete blood count and multiplex cytokine analysis of the blood. On the 7th day, there was a decrease in the overall leucocyte count caused by a decrease in neutrophil numbers, accompanied by reduced levels of certain interleukins in both experimental groups. This contrasts with the typical elevation in cytokine levels observed in human and rodent adenoviral infections or vaccinations [59].

Interestingly, following either the in vivo or ex vivo delivery of Ad5/35F-GFP, GFP-positive cells were detected only in immune system organs such as bone marrow, spleen, thymus, and submandibular lymph nodes, with no GFP-positive cells found in parenchymal organs like heart, lungs, liver, and kidneys. This suggests that the cells of parenchymal organs exhibit low sensitivity to the adenoviral chimeric vector after direct infusion of Ad5/35F-GFP and that ex vivo transduced leucocytes did not migrate to normal healthy organs. In contrast, the migration of gene-modified autologous leucocytes into a damaged brain after ischemic stroke or contusion injury was demonstrated in our previous studies [35,36,37].

Another important issue regarding parenchymal organs is the confirmation of a normal histology of the heart, lungs, liver, and kidneys, consistent with biochemical, and functional blood tests and indicates the absence of cytotoxicity effects in studied organs. Together with behavioral and instrumental data, these results suggest that Ad5/35F-GFP does not induce adverse effects following in vivo or ex vivo intravenous infusion in mini-pigs.

Recently, we developed a simple, safe, and economical approach for personalized gene therapy [37]. The gene engineered cells can be prepared at a hospital blood bank within a few hours in the blood container without the use of any substances and equipment for cell cultivation. To prepare such gene product, a certain amount of blood is taken from the patient; leucoconcentrate is isolated by centrifugation, into which chimeric adenoviral vector (Ad5/35F) or their combination carrying cDNA of the therapeutic genes is injected. The therapeutic effect of the leucoconcentrate enriched with genetic material will depend on personally selected therapeutic genes to be delivered into the patient’s leucocytes. The proposed method opens real prospects for the effective management of patients with stroke, neurotrauma, and bone fracture complications, as well as for stimulating vascular growth in cardiac and skeletal muscle ischemia, activating immunity in infections, etc. The possibility of freezing, storage, and subsequent use of the autologous gene-modified leucocytes will allow them to be procured in a personalized manner for persons with increased health risk (military, employees of the Ministry of Internal Affairs and the Ministry of Emergency Situations, athletes, etc.). The new platform can be adapted for implementation in obstetric clinics. Autologous leucocytes enriched with appropriate therapeutic genes can be prepared from cord blood mononuclear cells (cord blood collecting for banking is well established in many countries around the world) in maternity hospitals and used for personalized therapy of newborns with birth trauma, ischemic, infectious, and other diseases. Personalized gene therapy using patients’ leucocytes enriched with specially selected therapeutic genes for transient expression may become one of the breakthrough directions in gene therapy and may become an effective means of treatment and prevention of the most common diseases and cause social effects.

The aim of this study was to evaluate the safety and immune response to chimeric adenoviral vector Ad5/35F in large animal models. Analysis of comprehensive in vitro and in vivo studies showed no adverse effects and confirmed the immune tolerance of mini-pigs to Ad5/35F-GFP after direct or autologous leukocyte-mediated administration. The preliminary results of this pilot study suggest the need for further investigation with a larger cohort of mini-pigs and to include a shorter time point (1–3 days after the Ad5/35F administration) to evaluate the immediate innate immune response and the longer experimental period, which could be extended to approximately 2–3 weeks, to assess the adaptive immune response, and to examine the reaction of immunized animals to re-injection of the vector. While the low immune response in Vietnamese pot-bellied mini-pigs Ad5/35F-GFP is advantageous for minimizing side effects and ensuring effective transgene expression, the limited efficiency of porcine leucocyte transduction remains a concern. First, human adenoviruses have species-specific phenotypes [19] and, as mentioned above, even porcine adenoviruses are low-grade pathogens with low immunogenicity to the host [57]. Therefore, more studies are needed to improve the efficiency of porcine cell transduction with increasing MOI and using specific chemicals [60,61], which could improve the results and make the Vietnamese pot-bellied mini-pig model more robust.

Overall, this study provides fundamental insights into the biosafety of chimeric adenoviral vectors (Ad5/35F) for both direct and cell-mediated gene therapies, offering a valuable foundation for future research. We believe the results of this work make us close to our approach for personalized precision ex vivo gene therapy based on autoinfusion of the genetically enriched leucoconcentrate prepared from patient peripheral blood using a chimeric Ad5/35F viral vector for delivery therapeutic genes into the leucocytes.

## 5. Conclusions

The biosafety of gene therapy products is a critical concern in translational medicine. Immune responses to viral vectors, commonly used in the development of genetic therapies, can diminish the therapeutic efficacy of transgenes. The aim of this study was to evaluate the safety and immune response to the chimeric adenoviral vector Ad5/35F in large animal models with anatomical and physiological characteristics similar to those of humans. The results of in vitro transcriptome and secretome profiling of mini-pig leucocytes transduced with Ad5/35F carrying a GFP reporter gene suggest that the gene-modified leucocytes may have a low sensitivity to Ad5/35F. In in vivo studies, using behavioral, instrumental, and laboratory tests, we demonstrated that both the direct intravenous administration of Ad5/35F-GFP and the intravenous infusion of autologous leucocytes transduced with Ad5/35F-GFP caused no adverse effects related to immune activation. The observed immune tolerance of mini-pigs to Ad5/35F highlights the advantage of this vector system, as it minimizes side effects while enabling efficient transgene expression and evaluation of therapeutic efficacy. Therefore, we believe our data offer new fundamental insights into the safety of Ad5/35F and provide a solid platform for future clinical trials of autologous genetically enriched leucoconcentrate in ex vivo personalized gene therapy.

## Figures and Tables

**Table 1:** Primers and probes for RT-PCR.

Primer	Nucleotide Sequence
GFP-TM-Forward	AGCAAAGACCCCAACGAGAA
GFP-TM-Reverse	GGCGGCGGTCACGAA
GFP-TM-Probe	[FAM]CGCGATCACATGGTCCTGCTGG[BH1]
18S-TM-Forward	GGGAGGTAGTGACGAAAAATAACAAT
18S-TM-Reverse	TTGCCCTCCAATGGATCCT
18S-TM-Probe	[HEX]CGAGGCCCTGTAATTGGAATGAGTCCACT[BH2]

**Table 2:** Behavioral test results.

Experimental Groups	In Vivo								Ex Vivo							
Day of Study	0	1	2	3	4	5	6	7	0	1	2	3	4	5	6	7
Mean number of lines crossed (*n*) ^1^	****	**	**	*	*	**	*	**	*	***	**	***	***	**	**	***
Mean time of interaction with the ball (s) ^2^	+	+++	+	+++	++	+	++	+	+	+	+++	+	++++	+	++++	++++
Defecation	no	no	no	yes	yes	no	yes	yes	no	no	yes	no	yes	yes	no	yes
Urination	yes	no	no	no	yes	yes	no	yes	no	yes	no	no	no	no	no	yes

^1^ Mean number of lines crossed (*n*): * 1–6; ** 6–11; *** 11–16; **** 16–21. ^2^ Mean time of interaction with the ball (sec): + 0–100; ++ 100–200; +++ 200–300; ++++ ≥300.

**Table 3:** Blood test results.

Experimental Groups	In Vivo		Ex Vivo	
Day of Study	Day 0	Day 7	Day 0	Day 7
Leucocytes (10^9^/L)	15.20 ± 2.41	13.14 ± 0.80	16.76 ± 3.62	11.93 ± 1.37 *
Lymphocytes (10^9^/L)	7.20 ± 1.50	8.53 ± 1.56	6.95 ± 3.24	6.68 ± 1.48
Monocytes (10^9^/L)	0.49 ± 0.36	0.42 ± 0.50	0.25 ± 0.08	0.07 ± 0.02
Neutrophils (10^9^/L)	7.51 ± 0.95	4.20 ± 1.09	9.57 ± 4.86	5.18 ± 2.56 *
Total protein (g/L)	59.00 ± 6.08	59.00 ± 5.29	52.33 ± 4.04	52.33 ± 4.93
Albumin (g/L)	43.33 ± 1.15	42.00 ± 3.61	38.33 ± 2.52	36.67 ± 4.62
Globulin (g/L)	15.33 ± 4.73	17.00 ± 2.00	13.67 ± 1.53	15.33 ± 0.58
Calcium (mmol /L)	2.74 ± 0.26	2.50 ± 0.08	2.80 ± 0.22	2.50 ± 0.12
Sodium (mmol /L)	138.00 ± 1.73	140.00 ± 1.00	137.67 ± 0.58	138.00 ± 1.00
Potassium (mmol/L)	5.40 ± 0.20	5.40 ± 0.20	5.50 ± 0.17	5.37 ± 0.29
Chlorine (mmol/L)	94.67 ± 5.13	99.00 ± 1.00 *	93.67 ± 4.04	100.00 ± 1.73 *
Urea (mmol/L)	4.00 ± 0.17	2.53 ± 0.38 *	4.60 ± 0.75	2.90 ± 0.17 *
Creatinine (µmol/L)	53.33 ± 16.65	44.33 ± 25.01	61.00 ± 6.93	38.00 ± 9.17
Alanine Aminotransferase (U/L)	27.67 ± 8.96	35.00 ± 4.00	29.67 ± 9.45	30.00 ± 7.55
Total bilirubin (mmol/L)	4.00 ± 0.00	4.67 ± 1.15	4.00 ± 0.00	4.00 ± 0.00
Glucose (mmol/L)	5.07 ± 2.35	4.67 ± 0.58	4.43 ± 0.72	5.07 ± 0.25

* *p* < 0.05.

## Data Availability

The original contributions presented in the study are included in the article/Supplementary Material, further inquiries can be directed to the corresponding authors.

## Supplementary Materials

The following supporting information can be downloaded at: https://www.mdpi.com/article/10.3390/biomedicines12112568/s1, Table S1: The list of genes considered as differentially expressed NTC vs GFP; Table S2: The functional evaluation of the differentially expressed genes based on g:Profiler.
